# Prevention of non‐alcoholic steatohepatitis by long‐term exercise via the induction of phenotypic changes in Kupffer cells of hyperphagic obese mice

**DOI:** 10.14814/phy2.14859

**Published:** 2021-05-15

**Authors:** Ikuru Miura, Shoichi Komine, Kosuke Okada, Shota Wada, Eiji Warabi, Fumihiko Uchida, Sechang Oh, Hideo Suzuki, Yuji Mizokami, Junichi Shoda

**Affiliations:** ^1^ Graduate School of Comprehensive Human Sciences University of Tsukuba Tsukuba‐shi Ibaraki Japan; ^2^ Faculty of Human Care Department of Acupuncture and Moxibustion Teikyo Heisei University Toshima‐ku Tokyo Japan; ^3^ Faculty of Medicine University of Tsukuba Tsukuba‐shi Ibaraki Japan; ^4^ Tsukuba Preventive Medicine Research Center University of Tsukuba Hospital Tsukuba‐shi Ibaraki Japan; ^5^ Division of Biomedical Sciences Faculty of Medicine University of Tsukuba Tsukuba‐shi Ibaraki Japan

**Keywords:** dehydroepiandrosterone, Kupffer cell, long‐term exercise, non‐alcoholic fatty liver disease

## Abstract

Exercise ameliorates nonalcoholic fatty liver disease (NAFLD) by inducing phenotypic changes in Kupffer cells (KCs). *p62*/*Sqstm1*‐knockout (*p62*‐KO) mice develop NAFLD alongside hyperphagia‐induced obesity. We evaluated (1) the effects of long‐term exercise on the foreign‐body phagocytic capacity of KCs, their surface marker expression, and the production of steroid hormones in *p62*‐KO mice; and (2) whether long‐term exercise prevented the development of non‐alcoholic steatohepatitis (NASH) in *p62*‐KO mice fed a high‐fat diet (HFD). In experiment 1, 30‐week‐old male *p62*‐KO mice were allocated to resting (*p62*‐KO‐Rest) or exercise (*p62*‐KO‐Ex) groups, and the latter performed long‐term exercise over 4 weeks. Then, the phenotype of their KCs was compared to that of *p62*‐KO‐Rest and wild‐type (WT) mice. In experiment 2, 5‐week‐old male *p62*‐KO mice that were fed a HFD performed long‐term exercise over 12 weeks. In experiment 1, the phagocytic capacity of KCs and the proportion of CD68‐positive cells were lower in the *p62*‐KO‐Rest group than in the WT group, but they increased with long‐term exercise. The percentage of CD11b‐positive KCs was higher in the *p62*‐KO‐Rest group than in the WT group, but lower in the *p62*‐KO‐Ex group. The circulating dehydroepiandrosterone (DHEA) concentration was higher in *p62*‐KO‐Ex mice than in *p62*‐KO‐Rest mice. In experiment 2, the body mass and composition of the *p62*‐KO‐Rest and *p62*‐KO‐Ex groups were similar, but the hepatomegaly, hepatic inflammation, and fibrosis were less marked in *p62*‐KO‐Ex mice. The DHEA concentration was higher in *p62*‐KO‐Ex mice than in WT or *p62*‐KO‐Rest mice. Thus, long‐term exercise restores the impaired phagocytic capacity of KCs in NAFLD obese mice, potentially through greater DHEA production, and prevents the development of NASH by ameliorating hepatic inflammation and fibrogenesis. These results suggest a molecular mechanism for the beneficial effect of exercise in the management of patients with NAFLD.

## Graphical abstract

Long‐term running exercise by a high fat diet‐fed hyperphagic obese mice (*p62*‐KO mice) prevented the development of hepatic inflammation and fibrosis in non‐alcoholic steatohepatitis, via the restoration of foreign‐body phagocytic capacity and induction of phenotypic changes in the Kupffer cells.

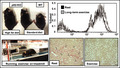

## 1 INTRODUCTION

Nonalcoholic fatty liver disease (NAFLD) is the most common chronic liver disease (Eguchi et al., 2012), and can be categorized histologically as nonalcoholic fatty liver (NAFL) or non‐alcoholic steatohepatitis (NASH). NAFL is defined as hepatic steatosis without evidence of hepatocellular injury, that is, simple steatosis; whereas, NASH is defined as hepatic steatosis and inflammation with hepatocyte injury, with or without fibrosis (Chalasani et al., 2018).

The development of NASH is not well understood; however, the multiple parallel hits hypothesis, in which multiple factors damage the liver in parallel with the development and progression of NASH, has been proposed to explain this (Tilg & Moschen, 2010). Lipopolysaccharide (LPS), a molecule derived from intestinal bacteria, is thought to play an important role in the pathogenesis of NASH (Schnabl & Brenner, 2014). In patients with NASH, LPS can easily enter the portal vein because of greater intestinal epithelial permeability, which leads to metabolic hyperendotoxemia (Wigg et al., 2001). The excess circulating LPS is thought to bind to toll‐like receptors (TLRs) on Kupffer cells (KCs), which are hepatic macrophages. This induces the innate immune response, resulting in the progression of steatosis and fibrosis in the liver, which then leads to NASH. Thus, the ability of KCs to process LPS appears to be important in the development and progression of NASH.

KCs are the resident macrophages of the liver, and account for approximately 80% of the total macrophage population of the body (Bouwens et al., 1986). KCs phagocytize foreign materials, such as LPS, as a part of the first step of the innate immune response (Mathison & Ulevitch, 1979; Smedsrod et al., 1994), and this phagocytosis is regulated by hormones and cytokines (Imajo et al., 2012). The foreign‐body phagocytic capacity of KCs has been reported to be lower in NAFLD in both humans (Shida et al., 2018) and animal models (Tsujimoto et al., 2008). Thus, abnormal phagocytosis by KCs appears to be involved in the progression of NAFLD to NASH, through a reduction in the clearance of LPS and the activation of inflammatory responses in KCs by the excess, which involve increases in inflammatory cytokine production and a wider increase in inflammation.

The results of a clinical study of exercise therapy in middle‐aged obese men conducted at our university indicated that an increase in moderate‐to‐high‐intensity exercise reduces hepatic fat accumulation and ameliorates inflammation, and oxidative stress (Oh et al., 2015). Furthermore, in a randomized controlled study, the phagocytic capacity of KCs was found to be improved by long‐term high‐intensity aerobic exercise (Oh et al., 2017).

In animal models, acute exercise has been reported to affect the kinetics of steroid hormones (Tatara & Sato, 2019; Yano et al., 2004) and increase the phagocytic capacity of KCs (Yano et al., 2004). We previously found that long‐term exercise potentiates the phagocytic capacity of KCs, increases the clearance of exogenously administered LPS, and reduces LPS‐induced inflammatory responses. We also demonstrated effects of exercise‐induced changes in dehydroepiandrosterone (DHEA) concentration on the phagocytic capacity of KCs and the inflammatory response induced by LPS in cell‐based experiments (Komine et al., 2017).

Mice with a deletion of *p62*/*Sqstm1* (*p62*‐KO), a regulator of selective autophagy, show hyperphagia‐induced obesity because of abnormal leptin signaling, and exhibit phenotypes similar to those of patients with NAFLD, including insulin resistance, impaired glucose tolerance, and NAFL (Harada et al., 2013; Okada et al., 2009). Thus, this mouse model exhibits hepatic pathology that includes inflammation and fibrosis; these both worsen if the mice consume a high‐fat diet (HFD). These features closely resemble the pathogenesis of NASH in humans (Duran et al., 2016).

In the present study, we have analyzed the function of KCs and evaluated the effects of long‐term exercise on the phagocytic capacity of these cells, their expression of surface markers, and the secretion of steroid hormones in *p62*‐KO mice that spontaneously develop NAFL in association with hyperphagia‐induced obesity (Harada et al., 2013; Okada et al., 2009). Furthermore, we have determined whether long‐term exercise can prevent the development of NASH, a pathological condition of the liver that is characterized by inflammation and fibrogenesis, and whether this might be through a potentiation of KC function, using *p62*‐KO mice fed a HFD (Duran et al., 2016).

## 2 MATERIALS AND METHODS

### 2.1 Experiment 1 protocol

In this experiment, two types of mice were used: 30‐week‐old male C57/BL6J mice, purchased from Charles River Japan, which were used as wild‐type (WT) mice, and 30‐week‐old male *p62*‐KO mice, which exhibited obesity with simple fatty liver, similar to that described in previous reports (Harada et al., 2013; Okada et al., 2009). The mice were housed under 12‐h light/dark cycle at 22.5 ± 1.4°C and 55.6% ± 4.0% relative humidity. The mice were allowed free access to food and water. After preliminary housing for 1‐week, they were allocated to three groups: a WT group, 1‐month resting *p62*‐KO group (*p62*‐KO‐Rest), and 1‐month exercising *p62*‐KO group (*p62*‐KO‐Ex), which was made to run on treadmill (MK‐680, Muromachi Kikai). Initially, there was 3‐day period of acclimation to the running, which involved 10 min of exercise at each of 5 and 10 m/min on day 1; 10 min each at 5, 10, and 15 m/min on day 2; and 10 min each at 10, 15, and 20 m/min on day 3. After this, the exercise was performed at 10, 12, 14, and 16 m/min for 5 min each and at 18 m/min for 30 min (50 min total) five times weekly for 4 weeks.

### 2.2 Experiment 2 protocol

In experiment 2, 4‐week‐old male WT and *p62*‐KO mice were weaned between 4 and 5 weeks of age, then maintained for a further week, before being allocated to three groups: a resting WT group that consumed standard diet, a resting *p62*‐KO group that consumed a HFD (HFD‐fed *p62*‐KO‐Rest), and an exercising *p62*‐KO group that consumed a HFD (HFD‐fed *p62*‐KO‐Ex) for 12 weeks. The mice were allowed free access to food and drinking water. The HFD was a 60% high‐fat/high‐sucrose diet (Oriental Yeast). The exercise group was made to run on the treadmill for 12 weeks, as described in the protocol for Experiment 1.

The study was approved by the Animal Care and Use Committee at the University of Tsukuba and was conducted in accordance with the University of Tsukuba Regulations on Animal Care and Use in Research; the Welfare and Management of Animals Act; and the Standards for Husbandry, Housing, and Pain Relief of Experimental Animals.

### 2.3 Sampling

Samples were collected 24 h after the final exercise bout under anesthesia with isoflurane (50 mg/kg body mass). The mice were weighed, and their livers and epididymal fat were sampled. Blood was collected from the abdominal vena cava, centrifuged at 1500 g for 15 min, and then the supernatants were frozen. To estimate whole‐body skeletal muscle and fat mass, each anesthetized mouse was placed on a polystyrene foam bed, and microcomputed tomography (CT) images were obtained and analyzed (LaTheta LCT‐200, Hitachi Aloka Medical). Tissue and serum samples were stored at −80°C.

### 2.4 Immunohistochemistry

The liver samples were washed with PBS, immersed in 4% paraformaldehyde for 24 h, dehydrated with 70% ethanol, and embedded in paraffin. The tissue was then cut into 5‐μm‐thick sections and stained with hematoxylin and eosin (H.E.) or Sirius Red. Based on the SAF score (Bedossa et al., 2012), the grade of liver pathology was determined by steatosis (from S0 to S3; lipid droplets S0: <5%, S1: 5–33%, S2: 34–66%, S3: >67%), activity (from A0 to A4 by adding grades of ballooning and lobular inflammation, both from 0 to 2; A0: no activity, A1: mild activity, A2: moderate activity, A3 (A ≥ 3): severe activity), and fibrosis (from F0 to F4; F0: none, F1: perisinusoidal zone or portal fibrosis, F2: perisinusoidal and periportal fibrosis without bridging, F3: bridging fibrosis, F4: cirrhosis). The evaluation of steatosis and activity was performed by H.E. staining, and the evaluation of fibrosis was performed by Sirius Red staining. The evaluation was carried out blinded by a skilled specialist. To quantitatively show the degree of liver fibrosis, the Sirius Red staining‐positive area in the lobus hepatis sinister was calculated. An all‐in‐one fluorescence microscope (BZ‐X800, Keyence) was used for imaging together with an analysis software (BZ‐H4C).

### 2.5 Isolation and functional analysis of Kupffer cells

To analyze the foreign‐body phagocytic capacity of KCs, these cells were isolated from livers 1 day after the end of the exercise period, as described previously (Komine et al., 2017). Under anesthesia, each liver was perfused with liver perfusion medium (Gibco, MA) for 4 min and then with DMEM containing collagenase type 4 (Worthington Biochemical), trypsin inhibitor (Wako Chemical, Osaka, Japan), and HEPES (Dojindo) for 10 min. Then, the livers were removed, and the cells were isolated in Hepatocyte wash medium (Gibco). The cell suspension was centrifuged at 30*g* for 2 min to remove hepatic parenchymal cells, then the supernatant was centrifuged at 400*g* for 8 min, and the pellet, containing the KCs, was subjected to fluorescence‐activated cell sorting (FACS)analysis.

The phagocytic capacity of the isolated KCs was analyzed by flow cytometry. Latex beads (FluoSpheres^®^, Invitrogen, 1.0 μm diameter, carboxylate‐modified) were administered at 0.57 μl/g body mass via a tail vein, and KCs were isolated 5 min later. Then, the proportion of KCs that had phagocytosed the latex beads was assessed by measuring the fluorescence intensity of the beads in cells that were positive for F4/80, a KC surface marker.

The expression of the KC surface proteins that are involved in phagocytosis was analyzed by flow cytometry using APC‐conjugated anti‐F4/80 (17‐4801‐82, eBioscience), PerCP/Cy5.5‐conjugated anti‐CD68 (137010, BioLegend, SAN), PE anti‐mouse CD284 (toll‐like receptor 4; TLR4) (145403, BioLegend), APC‐eFluor anti‐mouse CD11b (47011280, eBioscience), PE anti‐mouse CD206 (141705, BioLegend), and PE anti‐mouse CD206 (141705, BioLegend) antibodies. A Gallios flow cytometer (Beckman Coulter) was used for the measurements and Kaluza software (ver 1.2, Beckman Coulter) was used for data analysis.

### 2.6 Blood biochemistry

Blood was collected from a tail vein before and after exercise to measure the blood lactate concentration, as an indicator of exercise intensity (Lactate Pro2 LT‐1730, Arklay). Serum lipopolysaccharide (LPS) was measured using a Glucoshield Buffer kit (Associates of Cape Cod Inc.). The serum aspartate aminotransferase (AST) and alanine aminotransferase (ALT) activities were measured using standard protocols (Oriental Yeast Co., Ltd.). Serum glucose was measured using a LabAssay Glucose kit (Wako) and serum insulin was measured using a Mouse Insulin ELISA kit (Morinaga). Serum DHEA was measured using a DHEA ELISA kit (Enzo Life Sciences Inc.) and serum corticosteroids were measured using a Corticosterone ELISA kit (Cayman Chemical Company). Serum myostatin was measured using a GDF‐8/Myostatin Quantikine ELISA kit (R&D Systems) and serum follistatin was measured using a Mouse Follistatin ELISA kit (Raybiotech Life Inc.). Absorbances were measured using a Varioskan microplate reader (Thermo Fisher Scientific).

### 2.7 Real‐time PCR

The expression of hepatic mRNAs was analyzed by qPCR, as described previously (Akiyama et al., 2018). RNA was extracted from each liver sample and cDNA was synthesized using a PrimeScript RT reagent kit (Takara Bio). Then, qPCR was performed using the synthesized cDNA and SYBR Green Master Mix (Thermo Fisher Scientific). The mRNA expression levels of target genes were normalized to that of the gene encoding glyceraldehyde 3‐phosphate dehydrogenase (*Gapdh*). The primers used for qPCR are shown in Table 1.

**TABLE 1:** Primers used for quantitative real‐time PCR

Gene	Forward primer (5ʹ‐3ʹ)	Reverse primer (5ʹ‐3ʹ)
*Tnf‐α*	AAGCCTGTAGCCCACGTCGTA	GGCACCACTAGTTGGTTGTCTTTG
*Il‐1β*	TCCAGGATGAGGACATGAGCAC	GAACGTCACACACCAGCAGGTTA
*Tlr1*	CCCACAATGAGCTAAAGGTGATC	AGGCATTAAAGGAGAGGTCCAAA
*Tlr2*	CCCTTCTCCTGTTGATCTTGCT	CGCCCACATCATTCTCAGGTA
*Tlr4*	GCAGCAGGTGGAATTGTATCG	TGTGCCTCCCCAGAGGATT
*Tlr6*	AACCTTACTCATGTCCCCAAAGAC	GCATCCGAAGCTCAGATATAGAGTT
*Tlr9*	CCTTGACAACCTCCCCAAGA	AGGACTTCCAGGTTGGGTAGGA
*αSma*	ACCAACTGGGACGACATGGAA	TGTCAGCAGTGTCGGATGCTC
*Tgf‐β1*	GTGTGGAGCAACATGTGGAACTCTA	TTGGTTCAGCCACTGCCGTA
*Colla1*	GCACGAGTCACACCGGAACT	AAGGGAGCCACATCGATGAT

### 2.8 Cell experiments

We used a mouse macrophage‐like cell line (RAW264.7) and that with a deletion of *p62*/*Sqstm1* (*p62*‐KO), as described previously (Akiyama et al., 2018). These cells were cultured in high‐glucose DMEM (Nacalai Tesque) containing 10% fetal bovine serum (FBS), penicillin (50 U/ml), and streptomycin (50 mg/ml) in 5% CO_2_, and at 95% humidity and 37°C.

To analyze the phagocytic capacity of the RAW264.7 cells, 2.5 × 10^5^ cells were seeded in six‐well plates and incubated for 24 h. To assess phagocytic capacity, DHEA (TCI) in dimethyl sulfoxide (DMSO) was added at 10.0 ng/ml and then incubated for 12 h, after which latex beads in DMSO were added to the cells at 0.66 μl/ml and incubated for 12 h. The cells were then fixed in 4% paraformaldehyde and imaged using a fluorescence microscope. For flow cytometry analysis, the cells in the dish were washed twice and collected using a cell scraper. Next, the collected cells were further washed and centrifuged (4000 rpm for 3 min) twice, and then the fluorescence intensity of the beads in the cells was measured.

### 2.9 Statistical analysis

SPSS Statistics for Mac, version 26 (IBM, Inc.) was used for the statistical analyses. Data are expressed as mean ± standard error (SE). A paired *t*‐test was used to compare the blood lactate concentrations before and after exercise. Comparisons among three groups were performed using one‐way analysis of variance, followed by Tukey's multiple comparison test. A value of *p* < 0.05 was considered to represent statistical significance.

## 3 RESULTS

### 3.1 Effect of long‐term exercise on Kupffer cell function in mice with hyperphagia‐induced obesity and NAFL

**FIGURE 1:**
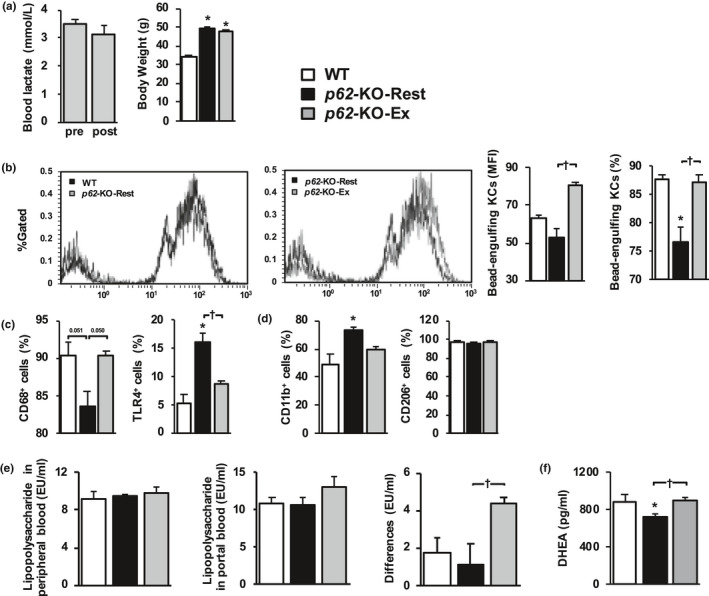
Long‐term exercise restores the foreign‐body phagocytic capacity of Kupffer cells (KCs) in *p62*‐KO mice. (a) Blood lactate concentration pre‐ and post‐exercise. The body mass of wild‐type mice fed a standard diet (WT, *n* = 3), resting *p62*‐KO mice fed a standard diet (*p62*‐KO‐Rest, *n* = 8), and *p62*‐KO fed a standard diet that exercised for 4 weeks (*p62*‐KO‐Ex, *n* = 8). (b) Latex bead‐phagocytic capacity of F4/80 positive cells (KCs) and their mean fluorescence intensity (MFI) in the livers of mice in each group. The MFI of the latex beads was determined using flow cytometry (*n* = 3/group). (c and d) Expression of the surface markers CD68 and toll‐like receptor (TLR) 4 (C), and CD11b (M1) and CD206 (M2) (d) in KCs from the livers of mice in each group (*n* = 3–4/group). (e) Lipopolysaccharide (LPS) concentrations in the peripheral and portal circulations and the differences between these (*n* = 5–6/group). (f) Dehydroepiandrosterone (DHEA) concentration in the peripheral blood (*n* = 6–8/group). Values are mean ± SE. *p < 0.05, compared with the WT group. ^†^ *p* < 0.05, compared with the *p62*‐KO‐Rest group

*p62*‐KO mice showed simple steatosis and no hepatic inflammation or fibrosis (data not shown), similar to the phenotype described in previous reports (Harada et al., 2013; Okada et al., 2009). Four weeks of long‐term exercise did not change the body mass or composition of the *p62*‐KO mice (Figure 1a). Furthermore, there was no change in blood lactate concentration during exercise, which implies that the exercise routine used in the study was of moderate or low‐intensity (Figure 1a). The changes in the foreign‐body phagocytic capacity of KCs were analyzed using flow cytometry (Figure 1b). Evaluation of the mean fluorescence intensity (MFI) of latex beads (the intensity of the right‐sided peak in the histogram) in F4/80‐positive cells (KCs) of cells from *p62*‐KO‐Ex mice was higher than that of the cells from *p62*‐KO‐Rest mice (Figure 1b). The proportion of KCs that had incorporated latex beads was higher in *p62*‐KO‐Ex mice than in *p62*‐KO‐Rest mice. The proportion in *p62*‐KO‐Ex mice was similar to that in WT mice (Figure 1b).

Analysis of the surface markers of KCs (Figure 1c and d) showed that the proportion of CD68‐positive cells was lower in *p62*‐KO‐Rest mice than in WT mice (*p* = 0.051), but higher in *p62*‐KO‐Ex mice (*p* = 0.050), which shows that it was increased by long‐term exercise. The proportion of TLR4‐positive cells was higher in *p62*‐KO‐Rest mice than in WT mice, but lower in *p62*‐KO‐Ex mice. The expression of proinflammatory (M1, CD11b) and anti‐inflammatory (M2, CD206) markers on the surfaces of KCs was also analyzed by flow cytometry. The results showed that M1 marker expression was lower in *p62*‐KO‐Ex mice than in *p62*‐KO‐Rest mice, whereas the level of M2 marker expression did not differ between the groups. No differences in the peripheral venous blood LPS concentration were identified among the WT, *p62*‐KO‐Rest, and *p62*‐KO‐Ex mice (Figure 1e). However, a difference in the LPS concentration was observed between the portal and peripheral blood samples from *p62*‐KO‐Ex mice (Figure 1e), which implies that the long‐term exercise increased the clearance of LPS by the liver. The serum DHEA concentration was higher in *p62*‐KO‐Ex mice than in *p62*‐KO‐Rest mice (Figure 1f).

**FIGURE 2:**
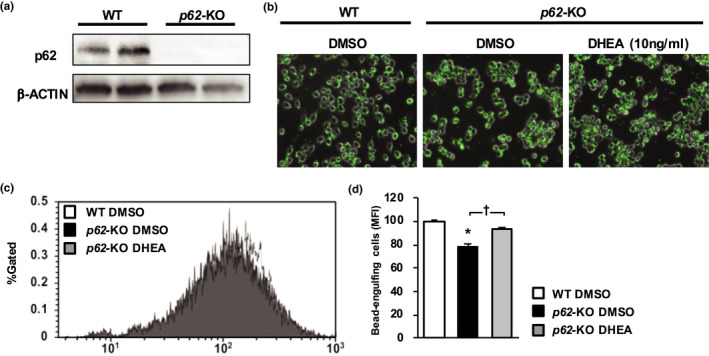
DHEA increases the foreign‐bodyphagocytic capacity of RAW264.7 cells. (a) Immunoblot analysis of p62 and β‐actin in RAW264.7 (WT) cells and those in which the *p62*‐gene was deleted (*p62*‐KO cells) using the CRISPR‐CAS9 system. (b–d) Latex beads in RAW264.7 cells and their mean fluorescence intensity (MFI) in WT and *p62*‐KO cells treated with dimethyl sulfoxide (DMSO) and in p*62*‐KO cells treated with dehydroepiandrosterone (DHEA). The latex beads are green. The MFI of the latex beads was determined using flow cytometry (*n* = 3/group). Values are mean ± SE. *p < 0.05, compared with the WT cells. ^†^ *p* < 0.05, compared with the *p62*‐KO cells treated with DMSO

The effect of DHEA on the phagocytic capacity of macrophages was determined in vitro. For this experiment, *p62*‐deficient RAW264.7 cells (*p62*‐KO‐RAW264.7) were prepared from a mouse macrophage‐like cell line (RAW264.7) using the CRISPR‐Cas9 system (Figure 2a). The MFI of the latex beads was higher in *p62*‐KO RAW264.7 cells that had been exposed to DHEA (10 ng/ml) than in cells that had been exposed to vehicle (DMSO) (Figure 2b–d).

### 3.2 Effects of long‐term exercise on the hepatic pathology of a mouse model of obesity and NASH

**FIGURE 3:**
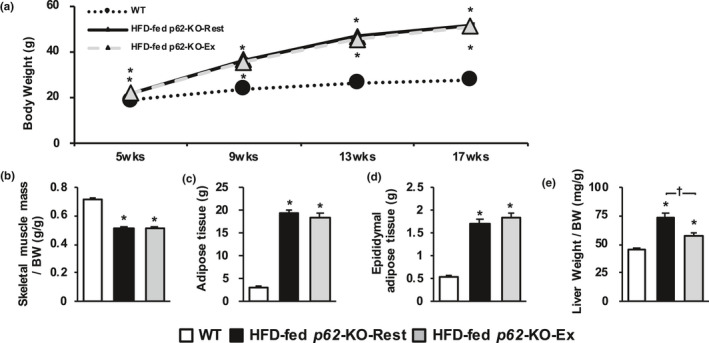
Long‐term exercise does not change body mass or composition but ameliorates the hepatomegaly of *p62*‐KO mice fed a high‐fat diet (HFD). (a) Time course of changes in body mass in wild‐type (WT) mice fed a standard diet (WT, *n* = 12), resting *p62*‐KO mice fed a HFD (HFD‐fed *p62*‐KO‐Rest, *n* = 12), and exercising *p62*‐KO mice fed a HFD (HFD‐fed *p62*‐KO‐Ex, *n* = 12). (b–e) Masses of skeletal muscle, the adipose tissue as a whole, epididymal adipose tissue, and the liver of mice in each group at 17 weeks of age (*n* = 11 or 12/group). Adipose and skeletal muscle masses were determined by computed tomography analysis. Values are mean ± SE. *p < 0.05, compared with the WT group. ^†^ *p* < 0.05, compared with the HFD‐fed *p62*‐KO‐Rest group

The consumption of a HFD‐induced obesity and NASH in the hyperphagic *p62*‐KO mice, and long‐term exercise had no effect on their body mass or composition (Figure 3a). Additionally, no effects were found on the total skeletal muscle mass or total body fat mass, assessed using CT, or the epididymal fat mass (Figure 3b–d) of these mice. However, long‐term exercise ameliorated the hepatomegaly of the mice (Figure 3e).

**FIGURE 4:**
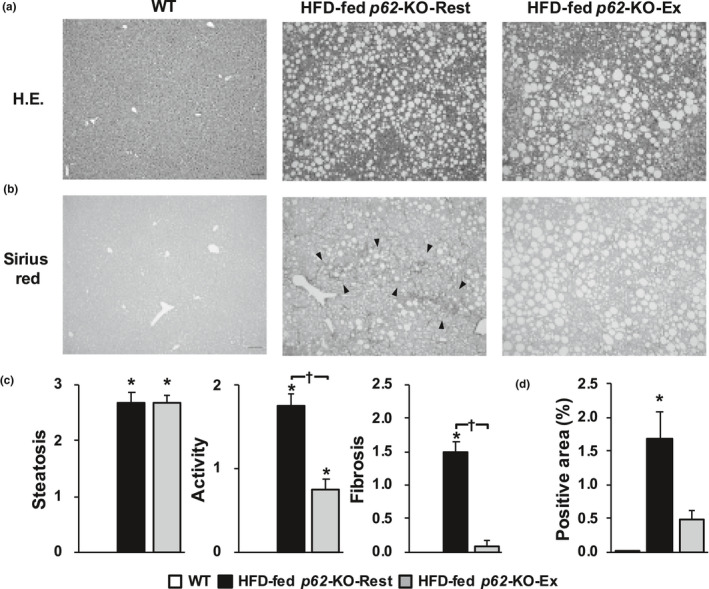
Long‐term exercise ameliorates the hepatic inflammation and fibrosis of *p62*‐KO fed a high‐fat diet (HFD). (a) Hematoxylin and eosin‐stained sections (scale bar, 100 μm) of livers from wild‐type (WT) mice fed a standard diet (WT), resting *p62*‐KO mice fed a HFD (HFD‐fed *p62*‐KO‐Rest), and exercising *p62*‐KO mice fed a HFD (HFD‐fed *p62*‐KO‐Ex). (b) Sirius Red‐stained sections (scale bar, 100 μm). The arrowheads point out a bridging fibrosis. (c) The steatosis, activity, and fibrosis (SAF) score (*n* = 12/group). (d) Area positive for Sirius Red staining (*n* = 10–22/group). Values are mean ± SE. *p < 0.05, compared with the WT group. ^†^ *p* < 0.05, compared with the HFD‐fed *p62*‐KO‐Rest group

The hepatic histopathology of the HFD fed *p62*‐KO mice was analyzed by determining the SAF score (Figure 4). This showed that long‐term exercise did not affect the degree of hepatic steatosis, but ameliorated the activity and fibrosis. When the degree of fibrosis was estimated on Sirius Red‐stained sections, the positive areas were smaller in HFD‐fed *p62*‐KO‐Ex mice than in HFD‐fed *p62*‐KO‐Rest mice, which implies that fibrosis is reduced by long‐term exercise.

**FIGURE 5:**
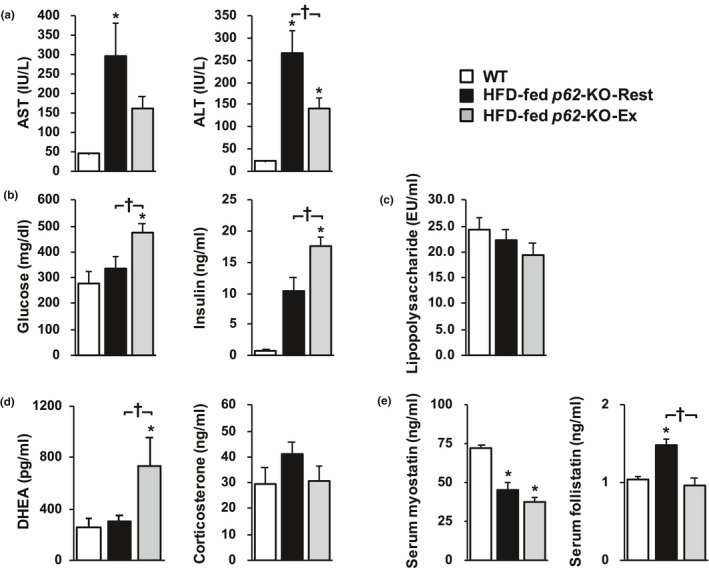
Blood biochemistry data. The circulating (a) AST and ALT, (b) glucose and insulin, (c) lipopolysaccharide, (d) DHEA and corticosterone, and (e) myostatin and follistatin concentrations were measured in wild‐type (WT) mice fed a standard diet (WT), resting *p62*‐KO mice fed a HFD (HFD‐fed *p62*‐KO‐Rest), and exercising *p62*‐KO mice fed a HFD (HFD‐fed *p62*‐KO‐Ex) (*n* = 6–14/group). Casual glucose and insulin concentrations were also measured. Values are mean ± SE. *p < 0.05, compared with the WT group. ^†^ *p* < 0.05, compared with the HFD‐fed *p62*‐KO‐Rest group. AST, aspartate aminotransferase; ALT, alanine aminotransferase; DHEA, dehydroepiandrosterone

The blood biochemistry analyses (Figure 5) showed that the serum AST and ALT activities were higher in the HFD‐fed *p62*‐KO‐Rest mice than in exercising mice, and the circulating glucose and insulin concentrations were higher in the HFD‐fed *p62*‐KO‐Ex mice. Although, no significant differences were found between the groups with respect to peripheral blood LPS concentration, long‐term exercise tended to ameliorate the metabolic hyperendotoxemia. The serum DHEA concentration of HFD‐fed *p62*‐KO‐Ex mice was higher than that in resting mice, whereas the blood corticosterone concentration did not differ between the two groups. Although, no difference was identified in the serum myostatin concentration between HFD‐fed *p62*‐KO‐Rest and *p62*‐KO‐Ex mice, the serum follistatin concentration was lower in the HFD‐fed *p62*‐KO‐Ex mice.

**FIGURE 6:**
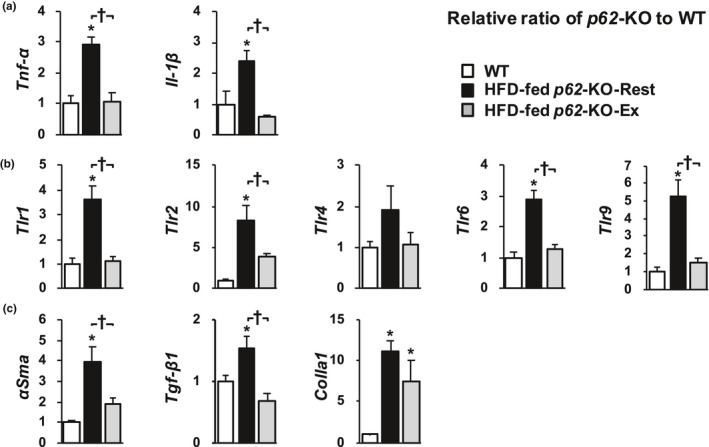
Long‐term exercise reduces proinflammatory signaling and the expression of fibrogenic mediators in the livers of *p62*‐KO mice fed a high‐fat diet (HFD). mRNA expression of (a) proinflammatory cytokines (*Tnf*‐*a* and *Il*‐*1β*), (b) toll‐like receptors (*Tlr1*, *2*, *4*, *6*, and *9*), and (c) fibrosis‐related genes (*αSma*, *Tgf*‐*β1*, and *Colla1*) (*n* = 9–10/group). Values are mean ± SE. *p < 0.05, compared with the WT group. ^†^ *p* < 0.05, compared with the HFD‐fed *p62*‐KO‐Rest group

qPCR analysis of the livers of the mice (Figure 6) showed that the expression of mRNAs for tumor necrosis factor‐α (*Tnf*‐*a*) and interleukin‐1β (*Il*‐*1β*) was lower in HFD‐fed *p62*‐KO‐Ex mice than in HFD‐fed *p62*‐KO‐Rest mice. The expression of genes encoding toll‐like receptors (*Tlr1*, *2*, *6*, and *9*) was lower in HFD‐fed *p62*‐KO‐Ex mice. Furthermore, the gene expression of α‐smooth muscle actin (*αSma*) and transforming growth factor‐1β (*Tgf*‐*β1*), which encode proteins involved in hepatic fibrosis, was lower in HFD‐fed *p62*‐KO‐Ex mice, but the expression of collagen type 1α (*Colla1*) was similar in the two groups.

## 4 DISCUSSION

In the present study, we have shown that the foreign‐body phagocytic capacity of KCs is attenuated in the hyperphagic *p62*‐KO mice, a mouse model of obesity and NAFL, and that long‐term exercise ameliorates this defect and improves LPS clearance by the liver. In vitro experiments showed that DHEA also improves the phagocytic capacity of *p62*‐KO macrophages. Thus, long‐term exercise in a HFD‐fed *p62*‐KO, a mouse model of obesity and NASH, mice ameliorates the hepatomegaly and hepatic inflammation and fibrosis, independent of any effect on body mass or composition. Furthermore, we have presented evidence that these effects are mediated through DHEA.

We have previously shown that obese patients with NAFLD, and particularly those with advanced hepatic fibrosis, frequently develop metabolic hyperendotoxemia, which is associated with a reduction in the phagocytic capacity of KCs and more severe inflammation and oxidative stress (Shida et al., 2018). Furthermore, the reduction in the phagocytic capacity of KCs is restored by long‐term high‐intensity exercise, which is followed by amelioration of the inflammation and oxidative stress (Oh et al., 2017). Thus, the present results are consistent with those of previous clinical studies.

The exercise program used in this study included an escalation at 0% inclination from 10 to 18 m/min, over 50 min, 5 days a week, which corresponded to moderate‐ or low‐ intensity exercise (Figure 1a). This is the exercise intensity that is commonly used for the prevention and treatment of obesity and lifestyle‐related diseases in humans (Oh et al., 2015). In the present study, long‐term exercise increased latex bead uptake by KCs in mice with hyperphagia‐induced obesity (Figure 1b–d). Few previous studies have reported the effects of exercise on the phagocytic capacity of KCs. Yano *et al*. reported that transient, acute exercise (15% inclination and 21 m/min for 60 min) by F344 rats increased the bead uptake capacity of their KCs and increased the LPS concentration in the portal vein (Yano et al., 2004). We previously reported that long‐term exercise by WT mice increases the uptake of fluorescent beads by KCs; that is, it potentiates their phagocytic capacity. It does not affect their body composition, the LPS concentration in the portal vein, or the number of KCs; but increases the clearance of exogenously administered LPS and limits inflammatory responses by inhibiting the LPS‐stimulated increases in inflammatory cytokine concentrations (Komine et al., 2017). Because these effects of exercise were absent in mice lacking KCs, the phenotypic changes in the KCs were considered to be caused by the exercise, and LPS stimulation alters the phagocytic capacity of KCs in vitro (Kinoshita et al., 2010).

Long‐term exercise potentiated bead uptake by the KCs of *p62*‐KO mice in the present study (Figure 1b). This increase in phagocytic capacity was attributed to the greater expression of the macrophage surface marker CD68 and scavenger receptors on KCs (Figure 1c). CD68‐positive KCs are a population of cells that have a high bead‐phagocytic capacity and may contribute to LPS clearance in the liver (Kinoshita et al., 2010). A long‐term exercise program is likely to increase the expression levels of these surface molecules in KCs, which would increase their phagocytic capacity.

We then evaluated the effects of long‐term exercise on hepatic inflammation and fibrosis in a mouse model of obesity and NASH, and found that long‐term exercise ameliorated both of these defects, independent of changes in body composition. This is important because the progression of hepatic fibrosis is a prognostic factor in human NASH (Loomba & Chalasani, 2015). The results of the present study suggest that long‐term exercise prevents the progression of NASH. Patients with NASH show signs of chronic inflammation, such as a high circulating LPS concentration (Wigg et al., 2001), low phagocytic capacity of KCs (Iijima et al., 2007), and hyperactivation of the innate immune response (Sharifnia et al., 2015). Long‐term exercise by mice with NAFL‐ induced phenotypic changes in their KCs and reduced the number of CD11b‐positive cells, which secrete proinflammatory cytokines. Furthermore, it reduced the number of cells that were positive for TLR4, a receptor for LPS (Figure 1). Long‐term exercise by mice with NASH reduced the hepatic expression of *Tlr* and the genes encoding the proinflammatory cytokines *Il*‐*1β* and *Tnf*‐*a* (Figure 6a and b), which suggests that long‐term exercise reduces the LPS‐induced inflammatory response in these mice. Additionally, the gene expression of *αSma* and *Tgf*‐*β1*, which induce hepatic fibrosis, was also reduced by long‐term exercise (Figure 6c). However, weight loss was not induced by the long‐term exercise (Figure 3a). These results suggest that inhibition of the NASH‐associated inflammation and fibrosis may occur secondary to increases in the phagocytic capacity of KCs and the phenotypic changes induced by long‐term exercise.

In previous studies of animal models of NASH, long‐term exercise has been reported to reduce the population of inflammatory (M1) macrophages in the liver (Gehrke et al., 2019; Kawanishi et al., 2012) and contribute to the inhibition of hepatic inflammation and fibrosis (Kawanishi et al., 2012). The results of the present study are consistent with those of previous studies and suggest that the increase in the phagocytic capacity of the KCs and the reduction in the inflammatory response form part of the mechanism whereby long‐term exercise reduces the number of M1 macrophages and inflammation.

We also evaluated factors that might mediate the alteration in the phenotype of KCs following long‐term exercise. When the peripheral concentrations of steroid hormones (Bongiovanni et al., 2015; Cao et al., 2019), which have been reported to affect phagocytic capacity, were measured in the present and previous studies, long‐term exercise was found to increase the DHEA concentration, but not that of corticosterone (Figure 5d). It has previously been reported (Komine et al., 2017; Tatara & Sato, 2019) that the serum DHEA concentration increases with exercise, and the half‐life of DHEA has been reported to be 7–22 hours (Rabe et al., 2015), which is longer than that of other hormones, which suggests that long‐term exercise would increase the circulating DHEA concentration.

When LPS was administered to mice after pretreatment with DHEA, their survival rate increased and TNF‐α secretion was inhibited (Danenberg et al., 1992). This suggests that long‐term exercise ameliorates inflammation and fibrosis in the liver by increasing the circulating DHEA concentration, which reduces the inflammatory response of KCs to LPS and the production of proinflammatory cytokines in the liver. Furthermore, in humans, a negative correlation has been reported between the hepatic pathology (steatosis and fibrosis) associated with NAFLD and the concentration of DHEA (Charlton et al., 2008).

We also assessed the in vitro effect of DHEA on the phagocytic capacity of RAW264.7 cells (KCs), and found it potentiated the phagocytic capacity of *p62*‐deficient KCs. Previous studies (Ben‐Nathan et al., 1999; Komine et al., 2017) have also shown that DHEA inhibits the LPS‐stimulated secretion of TNF‐α and the LPS‐induced inflammatory response in KCs, and the present findings are consistent with the findings of this study.

There are limitations to be overcome in this study. First, because we did not conduct DHEA administration experiments, we could not directly show the effect of DHEA on KCs function in vivo. Further studies are needed to directly show the effect of DHEA on KCs in vivo. Second, because the WT mice used in this study were purchased from a commercial company, it is possible that the mice had a number of unknown phenotypes different from the WT littermate controls, for example, a different intestinal microbiome. Third, the onset and progression of NASH in *p62*‐KO mice might be influenced by not only increased LPS production in the intestines, the disturbed phagocytotic function of KCs, but also insulin resistance and hyperleptinemia associated with obesity, and disturbed autophagy and activation of hepatic stellate cells associated with the *p62* deletion. Because these factors were not determined in this study and the effects of exercise training on these factors are unknown, further studies are needed to verify the preventive effects of exercise training on NASH.

## 5 CONCLUSIONS

Long‐term exercise restores the poor phagocytic capacity of KCs and induces phenotypic changes in their surface molecules in a mouse model of hyperphagia‐induced obesity and NAFL. Furthermore, the long‐term exercise for 12 weeks inhibits the progression of hepatic inflammation and fibrosis in a mouse model of obesity and NASH, thereby preventing the development of NASH, independent of weight loss. These effects appear to be mediated by increases in the phagocytic capacity of KCs and LPS clearance and a reduction in the inflammatory response, which is characterized by changes in the expression of surface markers on KCs. These effects may be mediated by an exercise‐induced increase in the production of DHEA. Thus, the results of this study provide a molecular mechanism for the beneficial effects of exercise in the management of patients with NAFLD.

## CONFLICT OF INTEREST

The authors declare that they have no competing interests.

## AUTHOR CONTRIBUTIONS

*Conception and design of research*: Ikuru Miura, Shoichi Komine, Kosuke Okada, Eiji Warabi, and Junichi Shoda. *Performed experiments*: Ikuru Miura, Shoichi Komine, Kosuke Okada, Shota Wada, and Sechang Oh. *Analyzed data*: Ikuru Miura, Shoichi Komine, Kosuke Okada, and Shota Wada. *Interpreted results of experiments*: Ikuru Miura, Shoichi Komine, Kosuke Okada, Shota Wada, Fumihiko Uchida, Hideo Suzuki, Yuji Mizokami, and Junichi Shoda. *Prepared figures*: Ikuru Miura, and Kosuke Okada. *Drafted manuscript*: Ikuru Miura, Shoichi Komine, and Kosuke Okada. *Edited and revised manuscript*: Kosuke Okada, and Junichi Shoda. *Approved final version of manuscript*: Junichi Shoda.

## References

[phy214859-bib-0001] Akiyama, K. , Warabi, E. , Okada, K. , Ynagawa, T. , Ishii, T. , Kose, K. , Yokushige, K. , Ishige, K. , Mizokami, Y. , Yanagawa, K. , Onizawa, K. , Ariizumi, S. , Ymamoto, M. , & Shoda, J. (2018). Deletion of both p62 and Nrf2 spontaneously results in the development of nonalcoholic steatohepatitis. Experimental Animals, 67(2), 201–218. 29276215 10.1538/expanim.17-0112 PMC5955752

[phy214859-bib-0002] Bedossa, P. , Poitou, C. , Veyrie, N. , Bouillot, J. L. , Basdevant, A. , Paradis, V. , Tordjiman, J. , & Clement, K. (2012). Histopathological algorithm and scoring system for evaluation of liver lesions in morbidly obese patients. Hepatology, 56(5), 1751–1759. 10.1002/hep.25889 22707395

[phy214859-bib-0003] Ben‐Nathan, D. , Padgett, D. A. , & Loria, R. M. (1999). Androsetenediol and dehydroepiandrosterone protect mice against lethal bacterial infections and lipopolysaccharide toxicity. Journal of Medical Microbiology, 48, 425–431. 10229539 10.1099/00222615-48-5-425

[phy214859-bib-0004] Bongiovanni, B. , Mata‐Espinosa, D. , D'Attilio, L. , Leon‐Contreras, J. C. , Marquez‐Velasco, R. , Bottasso, O. , Hernandez‐Pando, R. , & Bay, M. L. (2015). Effect of cortisol and/or DHEA on THP1‐derived macrophages infected with Mycobacterium tuberculosis. Tuberculosis (Edinb), 95(5), 562–569. 10.1016/j.tube.2015.05.011 26099547

[phy214859-bib-0005] Bouwens, L. , Baekeland, M. , De Zanger, R. , & Wisse, E. (1986). Quantitation, tissue distribution and proliferation kinetics of Kupffer cells in normal rat liver. Hepatology, 6, 718–722. 3733004 10.1002/hep.1840060430

[phy214859-bib-0006] Cao, J. , Yu, L. , Zhao, J. , & Ma, H. (2019). Effect of dehydroepiandrosterone on the immune function of mice in vivo and in vitro. Molecular Immunology, 112, 283–290. 10.1016/j.molimm.2019.06.004 31228660

[phy214859-bib-0007] Chalasani, N. , Younossi, Z. , Lavine, J. E. , Charlton, M. , Cusi, K. , Rinella, M. , Harrison, S. A. , Brunt, E. M. , & Sanyal, A. J. (2018). The diagnosis and management of nonalcoholic fatty liver disease: Practice guidance from the American Association for the Study of Liver Diseases. Hepatology, 67(1), 328–357. 10.1002/hep.29367 28714183

[phy214859-bib-0008] Charlton, M. , Angulo, P. , Chalasani, N. , Merriman, R. , Viker, K. , Charatcharoenwitthaya, P. , Sanderson, S. , Gawrieh, S. , Krishan, A. , & Lindor, K. (2008). Low circulating levels of dehydroepiandrosterone in histologically advanced nonalcoholic fatty liver disease. Hepatology, 47(2), 484–492. 10.1002/hep.22063 18220286 PMC2906146

[phy214859-bib-0009] Danenberg, H. D. , Alpert, G. , Lusting, S. , & Ben‐Nathan, D. (1992). Dehydroepiandrosterone protects mice from endotoxin toxicity and reduces tumor necrosis factor production. Antimicrobial Agents and Chemotherapy, 36(10), 2275–2279. 10.1128/AAC.36.10.2275 1444309 PMC245489

[phy214859-bib-0010] Duran, A. , Hernandez, E. D. , Reina‐Campos, M. , Castilla, E. A. , Subramaniam, S. , Raghunandan, S. , Roberts, L. R. , Kisselava, T. , Karin, M. , Diaz‐Meci, M. T. , & Moscat, J. (2016). p62/SQSTM1 by binding to vitamin D receptor inhibits hepatic stellate cell activity, fibrosis, and liver cancer. Cancer Cell, 30(4), 595–609. 10.1016/j.ccell.2016.09.004 27728806 PMC5081228

[phy214859-bib-0011] Eguchi, Y. , Hyogo, H. , Ono, M. , Mizuta, T. , Ono, N. , Fujimoto, K. , Chayama, K. , Saibara, T. , & JSG‐NAFLD . (2012). Prevalence and associated metabolic factor of nonalcoholic fatty liver disease in the general population from 2009 to 2010 in Japan: A multicenter large retrospective study. Journal of Gastroenterology, 47(5), 586–595. 22328022 10.1007/s00535-012-0533-z

[phy214859-bib-0012] Gehrke, N. , Biedenbach, J. , Huber, Y. , Straub, B. K. , Galle, P. R. , Simon, P. , & Schattenberg, J. M. (2019). Voluntary exercise in mice fed an obesegenic diet alters the hepatic immune phenotype and improves metabolic parameters an animal model of life style intervention in NAFLD. Scientific Reports, 9, 4007. 30850619 10.1038/s41598-018-38321-9 PMC6408519

[phy214859-bib-0013] Harada, H. , Warabi, E. , Matsuki, T. , Yanagawa, T. , Okada, K. , Uwayama, J. , Ikeda, A. , Nakaso, K. , Kirii, K. , Noguchi, N. , Bukawa, H. , Siow, R. C. M. , Mann, G. E. , Shoda, J. , Ishii, T. , & Sakurai, T. (2013). Deficiency of p62/sequestosome 1 cause hyperphagia due to leptin resistance in the brain. Journal of Neuroscience, 33(37), 14767–14777. 24027277 10.1523/JNEUROSCI.2954-12.2013 PMC6705174

[phy214859-bib-0014] Iijima, H. , Moriyasu, F. , Tsuchiya, K. , Suzuki, S. , Yosida, M. , Shimizu, M. , Sasaki, S. , Nishiguchi, S. , & Maeyama, S. (2007). Decrease in accumulation of ultrasound contrast microbubbles in non‐alcoholic steahepatitis. Hepatology Research, 37(9), 722–730. 17559420 10.1111/j.1872-034X.2007.00130.x

[phy214859-bib-0015] Imajo, K. , Fujita, K. , Yoneda, M. , Nozaki, Y. , Ogawa, Y. , Shinohara, Y. , Kato, S. , Mawatari, H. , Shibata, W. , Kitani, H. , Ikejima, K. , Kirikoshi, H. , Nakajima, N. , Saito, S. , Maeyama, S. , Watanabe, S. , Wada, K. , & Nakajima, A. (2012). Hyperresponsivity to low‐dose endotoxin during progression to nonalcoholic steatohepatitis is regulated by leptin‐mediated signaling. Cell Metabolism, 16(1), 44–54. 10.1016/j.cmet.2012.05.012 22768838

[phy214859-bib-0016] Kawanishi, N. , Yano, H. , Mizokami, T. , Takahashi, M. , Oyanagi, E. , & Suzuki, K. (2012). Exercise training attenuates hapatic inflammation, fibrosis, and macrophage infiltration during diet induced obesity in mice. Brain, Behavior, and Immunity, 26(6), 931–941. 10.1016/j.bbi.2012.04.006 22554494

[phy214859-bib-0017] Kinoshita, M. , Uchida, T. , Sato, A. , Nakashima, M. , Nakashima, H. , Shono, S. , Habu, H. , Miyazaki, H. , & Seki, S. (2010). Characterization of two F4/80‐postive Kupffer cell subsets by their function and phenotype in mice. Journal of Hepatology, 53(5), 903–910. 20739085 10.1016/j.jhep.2010.04.037

[phy214859-bib-0018] Komine, S. , Akiyama, K. , Warabi, E. , Oh, S. , Kuga, K. , Ishige, K. , Togashi, S. , Yanagawa, T. , & Shoda, J. (2017). Exercise training enhances in vivo clearance of endotoxin and attenuates inflammatory responses by potentiating Kupffer cell phagocytosis. Scientific Reports, 7, 11977. 10.1038/s41598-017-12358- 28931917 PMC5607327

[phy214859-bib-0019] Loomba, R. , & Chalasani, N. (2015). The Hierarchical Model of NAFLD: Prognostic significance of histologic features in NASH. Gastroenterology, 149(2), 278–281. 10.1053/j.gastro.2015.06.016 26116800

[phy214859-bib-0020] Mathison, J. C. , & Ulevitch, R. J. (1979). The clearance, tissue distribution, and cellular localization of intravenously injected lipopolysaccharide in rabbits. The Journal of Immunology, 123, 2133–2143. 489976

[phy214859-bib-0021] Oh, S. , Shida, T. , Yamagishi, K. , Tanaka, K. , So, R. , Tsujimoto, T. , & Shoda, J. (2015). Moderate to vigorous physical activity volume is an important factor for managing nonalcoholic fatty liver disease: A Retrospective Study. Hepatology, 61(4), 1205–1215. 10.1002/hep.27544 25271091

[phy214859-bib-0022] Oh, S. , So, R. , Shida, T. , Matuo, T. , Kim, B. , Akiyama, K. , Isobe, T. , Okamoto, Y. , Tanaka, K. , & Shoda, J. (2017). High‐intensity aerobic exercise improves both hepatic fat content and stiffiness in sedentary obese men with nonalcoholic fatty liver disease. Scientific Reports, 7, 43029. 28223710 10.1038/srep43029 PMC5320441

[phy214859-bib-0023] Okada, K. , Yanagawa, T. , Warabi, E. , Yamastu, K. , Uwayama, J. , Takeda, K. , Utsunomiya, H. , Yoshida, H. , Shoda, J. , & Ishii, T. (2009). The alpha‐glucosidase inhibitor acarbose prevents obesity and simple steatosis in sequestosome 1/A170/p62 deficient mice. Hepatology Research, 39(5), 490–500. 19207582 10.1111/j.1872-034X.2008.00478.x

[phy214859-bib-0024] Rabe, T. , Ahrendt, H. J. , Albring, C. , Bachmann, A. , Bitzer, J. B. P. U. , Egarter, C. , Hinney, B. , Kentenich, H. K. K. , Merki Feld, G. , Merkle, E. , Mueck, A. O. , & Rossmanith, W. G. (2015). Dehydroepiandrosterone and its sulfate joint statement by the German Society for Gynecological Endocrinology and Reproductive Medicine (DGGEF) and the German Professional Association of Gynecologists (BVF). Journal Für Reproduktionsmedizin Und Endokrinologie, 12, 318–341.

[phy214859-bib-0025] Schnabl, B. , & Brenner, D. A. (2014). Interactions between the intestinal microbiome and liver diseases. Gatroenterology, 146, 1513–1524. 10.1053/j.gastro.2014.01.020 PMC3996054 24440671

[phy214859-bib-0026] Sharifnia, T. , Antoun, J. , Verriere, T. G. C. , Suarez, G. , Wattacheril, J. , Wilson, K. T. , Peek, R. M. Jr , Abumrad, N. N. , & Flynn, C. R. (2015). Hepatic TLR4 signaling in obese NAFLD. American Journal of Physiology, 309(4), 270–278. 10.1152/ajpgi.00304.2014 PMC4537925 26113297

[phy214859-bib-0027] Shida, T. , Akiyama, K. , Oh, S. , Sawai, A. , Isobe, T. , Okamoto, Y. , Ishige, K. , Mizokami, Y. , Yamagata, K. , Onizawa, K. , Tanaka, H. , Iijima, H. , & Shoda, J. (2018). Skeletal muscle mass to visceral fat area ratio is an important determinant affecting pathophysiology of NAFLD. Journal of Gastroenterology, 53, 535–547. 28791501 10.1007/s00535-017-1377-3

[phy214859-bib-0028] Smedsrod, B. , De Bleser, P. J. , Braet, F. , Lovisetti, P. , Vanderkerken, K. , Wisse, W. , & Geerts, A. (1994). Cell biology of liver endothelial and Kupffer cells. Gut, 35(11), 1509–1516. 10.1136/gut.35.11.1509 7828963 PMC1375602

[phy214859-bib-0029] Tatara, K. , & Sato, K. (2019). Aerobic exercise training and dehydroepiandrosterone administration increase testicular sex steroid hormones and enhance reproductive function in high‐sucrose‐induced obese rats. Journal of Steroid Biochemistry and Molecular Biology, 190, 37–43. 10.1016/j.jsbmb.2019.03.019 30923020

[phy214859-bib-0030] Tilg, H. , & Moschen, A. R. (2010). Evolution of inflammation in nonalcoholic fatty liver disease: The multiple parallel hits hypothesis. Hepatology, 52(5), 1836–1846. 10.1002/hep.24001 21038418

[phy214859-bib-0031] Tsujimoto, T. , Kawaratani, H. , Kitazawa, T. , Hirai, T. , Ohishi, H. , Kitade, M. , Yoshiji, H. , Uemura, M. , & Fukui, H. (2008). Decreased phagocytic activity of Kupffer cells in a rat nonalcoholic steatohepatitis model. World Journal of Gastroenterology, 14(39), 6036–6043. 10.3748/wjg.14.6036 18932283 PMC2760201

[phy214859-bib-0032] Wigg, A. J. , Roberts‐Thomson, I. C. , Dymock, R. B. , McCarthy, P. J. , Grose, R. H. , & Cummins, A. G. (2001). The role of small intestinal bacterial overgrowth intestinal permeability, endotoxaemia, and tumour necrosis factor α in the pathogenesis of non‐alcoholic steatohepatitis. Gut, 48(2), 206–211. 11156641 10.1136/gut.48.2.206 PMC1728215

[phy214859-bib-0033] Yano, H. , Kinoshita, S. , & Kira, S. (2004). Effect of acute moderate exercise on the phagocytosis of Kupffer cells in rat. 2004. Acta Physiologica Scandinavica, 182, 151–160. 15450111 10.1111/j.1365-201X.2004.01343.x

